# The Role of Conventional and Stereotactic Microwave Ablation for Intrahepatic Cholangiocarcinoma

**DOI:** 10.3390/jcm10132963

**Published:** 2021-07-01

**Authors:** Corina Kim-Fuchs, Daniel Candinas, Anja Lachenmayer

**Affiliations:** Department of Visceral Surgery and Medicine, University Hospital Bern, University of Bern, 3010 Bern, Switzerland; corina.kim-fuchs@insel.ch (C.K.-F.); daniel.candinas@insel.ch (D.C.)

**Keywords:** computer-assisted ablation, stereotactic ablation, intrahepatic cholangiocarcinoma, minimal-invasive liver treatments, microwave ablation

## Abstract

Background: The incidence and mortality of intrahepatic cholangiocarcinoma (ICCA) is increasing worldwide and curative treatment options are limited due to the aggressive tumor biology and often late diagnosis. Resection of the primary tumor remains the only curative therapy available, as the benefit of palliative chemotherapy and radiotherapy is relatively small. In contrast to hepatocellular carcinoma, minimal-invasive thermal tumor ablation, and in particular stereotactic tumor ablation for small primary cancers or metastases, is not established and data are scarce. Methods: We conducted a literature review in the field of ICCA ablation and retrospective analysis of 10 patients treated by stereotactic microwave ablation (SMWA) for either primary ICCA or liver metastases of ICCA. Results: While current guidelines have no consensus for ablation of primary ICCA, some state that it might be an option in inoperable patients or those with recurrent disease. The literature review revealed 11 studies on microwave ablation for ICCA reporting that MWA for ICCA ≤ 5 cm might be safe and could be a treatment option for patients who are not candidates for surgery. No data has been published on stereotactic microwave ablation (SMWA) for ICCA. The analyses of our own data of 10 patients treated by SMWA for primary ICCA (*n* = 5) or recurrent ICCA (*n* = 5) show that the treatment is safe and efficient with short hospital stays and low complication rates. Conclusion: Although thermal ablation, and in particular SMWA, might be a minimally invasive and tissue-sparing curative treatment alternative for small ICCA in the diseased liver and ICCA metastases, the oncologic benefit still needs to be shown in larger studies with longer follow-up.

## 1. Introduction

Cholangiocarcinoma (CCA) is the second most common liver cancer worldwide and accounts for about 15% of all primary liver tumors [1]. The incidence is increasing over the last decades with 0.3–6 cases per 100,000 people per year [2]. Intrahepatic cholangiocarcinomas (ICCA) arise distal to the second-order bile ducts and can be clearly distinguished from perhilar and hilar cholangiocarcinomas [3]. While most known risk factors for ICCA are in one way or another associated with chronic inflammation of the bile ducts, chronic liver disease (cirrhosis, viral hepatitis) and physiochemical irritation [4], and patient-related factors such as diabetes, obesity, smoking, hypertension and alcohol consumption have also been described [1,3]. The treatment of choice is surgery; however, a resection is only feasible in 20–40% of cases [5,6,7]. There is a high recurrence rate after a successful resection (60%) and the 5-year survival rate after surgery is only 20–44% if the tumor can be resected with a clear safety margin [8,9,10]. If the operation cannot be completed with clear margins, the 5-year survival drops to 0% [11]. In addition, patients with positive lymph nodes only have a 5-year survival of 0–9% compared with 43% in patients with no lymph node metastases [12]. Liver transplantation might be a treatment option in patients with limited disease and underlying cirrhosis, but data are rare and results from ongoing clinical trials are urgently awaited. While palliative treatments (chemotherapy, radiotherapy) have limited effect on these tumors with a median survival around 5–8 months [13,14], some new targeted treatment options show promising first results [15,16,17]. The therapeutic resistance of ICCA is based on the genetic heterogeneity, the highly desmoplastic nature and complex tumor microenvironment in the liver [18]. Thermal ablation, such as microwave ablation (MWA) and/or radiofrequency ablation (RFA), has become an important curative treatment option for hepatocellular carcinoma (HCC), but data for ICCA are very limited. Ablation has the advantage of being minimally invasive and tissue sparing, also in non-surgical candidates or those with underlying liver disease [19]. MWA seems to have several advantages over RFA including higher intra-tumoral temperatures, larger ablation volumes, shorter operation times and less dependence on the electrical conductivity of tissues [20,21].

Recently, computer-assisted navigation systems have been introduced to increase the efficacy and safety of liver tumor ablations and to increase the feasibility of tumor ablation for lesions in difficult to treat anatomic locations [19]. Several studies have now demonstrated the safe clinical application of these systems in particular for HCC and liver metastases, but not much is known for ICCA.

Herein, we aim to summarize the current literature on computer-assisted and conventional ICCA ablation and present our own clinical data of stereotactic microwave ablation (SMWA) for ICCA primary tumors and ICCA metastases.

## 2. Materials and Methods

**Figure 1:**
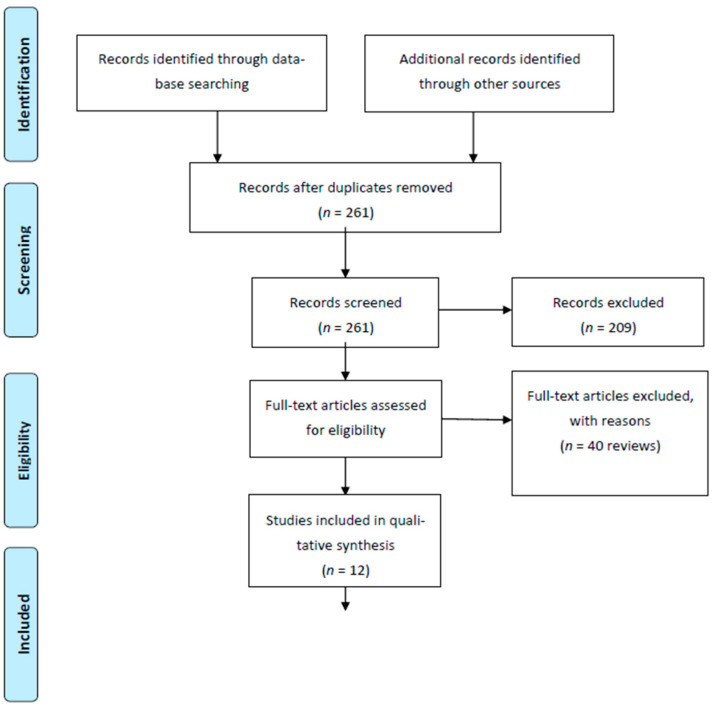
Flow diagram.

A systematic literature search was performed using PubMed, OVID MEDLINE, EMBASE, Scopus and Cochrane library with the mesh terms: intrahepatic cholangiocarcinomas (all fields) AND microwave ablation (all fields). We found 261 matches. All titles and abstracts were screened and after exclusion of papers not published in English, case reports, book chapters and letters, we excluded 65 review papers. We analyzed 12 papers (Figure 1).

## 3. Results

### 3.1. Current Guideline Recommendation for ICCA Ablation

Most of the current guidelines for the treatment for ICCA give no specific treatment recommendation for ICCA ablation. The European Society of Medical Oncology (ESMO) guidelines only mention radiotherapy and Y90 radioembolization for irresectable or locally advanced and non-metastatic tumors [22]. The British Society of Gastroenterology (BSG) guideline of 2012 suggests RFA as a safe treatment option for small, unresectable ICCA, but there are no treatment recommendations for the use of MWA. Instead, they report the results of a small Chinese study where 18 patients (8 primary and 10 recurrent cases after resection) were treated with ultrasound-guided thermal ablation with curative intention showing a complete ablation rate of 92% with an overall survival rate of 30% at 36 months [23]. The European Association for the Study of the Liver (EASL) guideline of 2014 state that a small group of patients with liver-only recurrence may be treated by either ablation or resection [24]. In addition, the latest National Comprehensive Cancer Network (NCCN) guideline from 2021 only gives a weak recommendation for loco regional therapies such as RFA and TACE in patients with unresectable tumors or metastatic cancer without extrahepatic disease [25]. Overall, there are no established first-line ablation options for patients with resectable and unresectable ICCA or ICCA metastases. Ablation approaches may be considered for small and single lesions in individual cases.

### 3.2. MWA for ICCA

Although MWA is an efficient therapy for HCC, this technique is not very common for the treatment of ICCA and only a few retrospective studies have been published (Table 1); therefore, evidence is limited compared with HCC or colorectal liver metastasis treated with MWA. Even though the selection bias in the published studies is high, most tumors were treated in curative intent and not in a palliative setting. In case of recurrence after ICCA resection, ablation was also performed in curative intent and most studies excluded patients with extrahepatic disease.

One of the first papers, investigating MWA for ICCA included 15 patients with primary ICCA [26]. The success rate of the treatment was 91.7% and the local tumor progression rate was 25%. The cumulative overall survival rate for 6, 12 and 24 months were 78.8%, 60.0% and 60.0%, respectively. Xu et al. published similar results after one year, including 18 patients: 8 with primary tumors and 10 with recurrent disease [23]. A few years later, in 2017, one of the largest studies looking at MWA in ICCA was published by Zhang et al. [28]. They included 107 patients with 171 lesions in curative intention. The lesions were smaller than 5 cm, and the number of lesions per patient was ≤3. The median PFS was in 8.9 months, the PFS rate after 6, 12, 18 and 24 months was 67.4%, 41.5%, 18.2% and 8.7%, respectively. The median overall survival was 28 months, and the OS rate after 1, 3 and 5 years was 93.5%, 39.6% and 7.9%, respectively. The group concluded that MWA for ICCA ≤5 cm is safe as a treatment option in patients who are not candidates for surgery. Two studies looked at the outcomes of ICCA after MWA based on Albumin–Bilirubin grade (ALBI) [31,36]. The ALBI was used as a predictive value for long-term overall survival after MWA in ICCA patients. The ALBI grade may offer a prognostic tool to evaluate patients for MWA in early ICCA. Additionally, in these studies the OS was similar to other studies (1-, 3- and 5-year OS: 87.4%, 51.4% and 35.2%, respectively). Most of the included studies did not specifically address the effect of neo-adjuvant or adjuvant chemotherapy. Yang et al. reported 11 patients who received an adjuvant therapy with gemcitabine and cisplatin after MWA but did not stratify the results accordingly [31]. Only the work by Takahashi et al. showed that a neo-adjuvant treatment with chemotherapy did not reduce the risk for local tumor progression [32].

### 3.3. Ablation vs. Surgery

There was one study comparing ultrasound-guided MWA vs. surgery retrospectively [35]. In this study, 121 consecutive patients were included, 56 with MWA (62 lesions) and 65 with surgical resection (74 lesions). Both techniques had a success rate of 100%. The difference in recurrence rates was not significant (9/56 vs. 8/65). There was also no difference in the 1-, 3- and 5-year OS. The authors concluded that ablation had the same efficacy as surgery for recurrence and should be preferred in patients with liver function of ALBI grade 2 [35]. 

### 3.4. RFA vs. MWA

There are several studies comparing RFA and MWA. In 2018 Takahashi et al. reported the results of a small retrospective study, analyzing 20 patients with 50 lesions [32]. They performed percutaneous RFA (44 patients) and only four MWA. The tumor size in both treatment groups was very small with a mean size of 1.8 cm. A larger, multicenter study was done in Italy, also comparing the two techniques. In total, 71 patients were treated, including 36 with RFA and 35 with MWA [29]. They concluded that patients treated with MWA for ICCA nodules <3 cm survived longer than patients treated with RFA (*p* < 0.005). The OS in the MWA group was 95%, 75%, 68% and 68% at 12, 36, 60 and 80 months, and 86%, 53%, 26% and 13% in the RFA group, respectively, (*p* < 0.005). Additionally, the progression-free survival was higher in the MWA group compared with the RFA group, with 79%, 59%, 55% and 55% at 12, 36, 60 and 80 months in the MWA group, respectively, and 69%, 51%, 8.5% and 8.5% at 12, 36, 60 and 80 months in the RFA group, respectively. Another study looked at the topic of thermal ablation for ICCA in cirrhosis [30]. They included patients with 27 patients with ICCA, Child A/B without ascites/encephalopathy and with cirrhosis and performed RFA or MWA. In conclusion, they reported that thermal ablation is also safe and effective in treatment for ICCA in patients with liver cirrhosis [30].

### 3.5. MWA and TACE

As known from HCC, MWA combined with transarterial tumor embolization results in a high complete response rate, in particular in larger tumors [37,38]. A group in China conducted a retrospective analysis of the efficacy of percutaneous microwave ablation combined with simultaneous transarterial chemoembolization (TACE) [39]. They included 26 patients in their study, including 20 primary tumors, and 6 with recurrence after surgery. The treated tumors were 3.6 ± 1.1 cm in patients with Child A or B. The median progression free survival was 6.2 months with an OS at 12 and 24 months of 69% and 62%, respectively. Another group from Ge et al. analyzed 275 patients with recurrent ICC comparing TACE vs. MWA. The treated tumors were 6.5 ± 3.7 for TACE and 5.7 ± 2.4 cm for MWA. In their study, TACE provided a higher survival benefit. 

### 3.6. Stereotactic Microwave Ablation (SMWA)

One study from Germany compared the efficacy of stereotactic MWA with conventional free-hand MWA of malignant liver tumors [40]. They included 221 patients with 30 patients with ICCA. While the stereotactic navigation improved primary efficacy compared to conventional guidance (84.3% vs. 75.0%), no specific data were given on the results of the treated ICCAs.

### 3.7. ICCA and Stereotactic MWA: Clinical Trials

Up until today, no randomized controlled or other clinical trials have been performed to analyze the efficacy of stereotactic MWA (SMWA) for ICCA. Currently, ClinicalTrials.gov has five trials registered for the treatment of ICCA (05/2021). While one is a registry, three are analyzing the combination of ablation with systemic treatments, one is studying RFA for all biliopancreatic malignancies and one is examining a novel RFA device for other liver malignancies as well. No study is currently ongoing analyzing the effect of SMWA or other computer-assisted navigation devices for the treatment for ICCA. 

### 3.8. Own Patients with ICCA Treated by SMWA 

**Figure 2:**
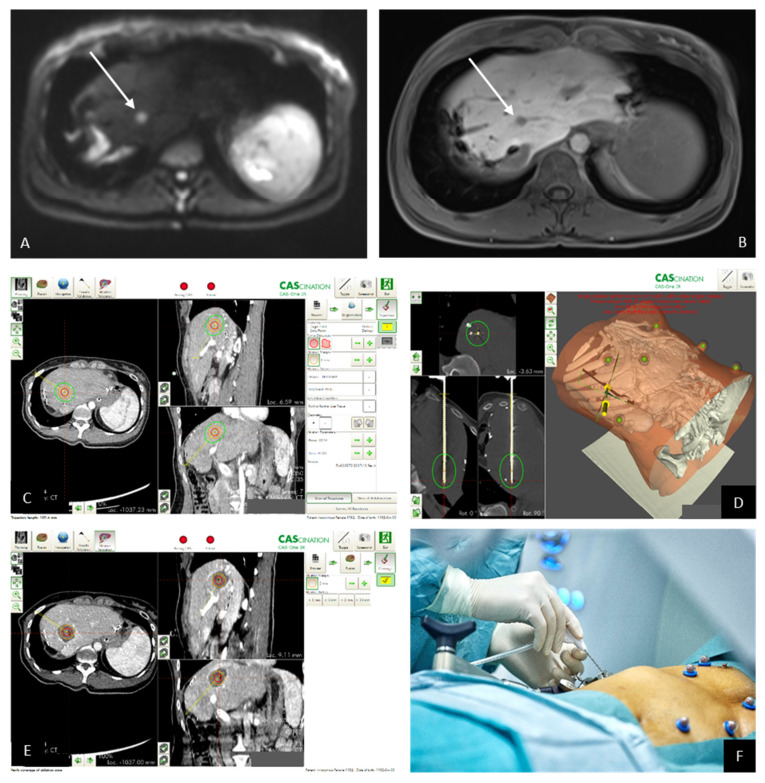
(**A**) Pre-interventional MRI imaging reveals the metastasis with high signal in the b800 image of the diffusion-weighted imaging (DWI) and also shows (**B**) a lack of intracellular uptake of hepatocyte-specific contrast medium in the hepatobiliary phase. (**C**) The pre-interventional planning of the ablation zone (red circle = segmented tumor; (**D**) green circle = anticipated ablation zone) needle validation scan with the needle in place and the anticipated ablation zone in green. (**E**) The ablation zone in the contrast-enhanced control scan; (**F**) needle in place.

During the past three years, we performed SMWA for 10 patients with ICCA. Five patients had primary ICCA and underlying liver disease, five patients had metastatic disease after previously resected ICCA in healthy livers. Table 2 summarizes the results of the five patients treated by SMWA for primary ICCA. While three patients were treated for a suspected HCC and had an incidental finding of ICCA in the biopsy, two patients were diagnosed before SMWA. One patient developed a local recurrence after 18 months and is now planned for a combined treatment of embolization and ablation. Another patient was transplanted 10 months after the ablation showing no disease recurrence in the explanted liver or afterwards. Table 3 gives an overview of the patients treated with SMWA for recurrent ICCA. While three patients are currently tumor free, two developed local recurrences and simultaneous overall disease progression leading to tumor-related mortality. One patient died without disease recurrence. Figure 2 gives an example of one patient with recurrent ICCA treated by SMWA. SMWA allows for a tumor segmentation and planning of the ablation zone, a precise placement of the ablation probe and an immediate validation of the ablation zone in respect to the tumor within the intervention. In case of invisibility of the tumor, the pre-interventional MRI images can be fused with the CT-scan for the treatment.

## 4. Discussion

MWA and SMWA are not established therapies for the treatment of ICCA. Up until now, only very limited data are available on the safety and efficacy of conventional image-guided ICCA ablation in general, and no specific data are available for the use of computer-assisted navigation approaches, in particular not for SMWA.

There is no consensus for the use of ablation for ICCA and current guidelines report different treatment recommendations [22,24,41]. While some recommend the use of ablation for irresectable or recurrent tumors, no guideline recommends ablation as a first line treatment. Due to the small number of reported cases of only retrospective nature, the evidence for all of these recommendations is rather small. 

Our literature research revealed 11 papers analyzing the effect of microwave ablation for ICCA showing that MWA is feasible and safe [23,26,28,31,36]. The studies were all done retrospectively with small patient numbers. MWA was used in patients with primary tumors, who are not surgical candidates or with recurrent tumors. Despite the small cohorts and the retrospective design of the studies, the general conclusion of these papers was that MWA might prolong the overall survival of patients and that it could be more effective than RFA. Overall, it seems that most of these studies focus on ICCAs developing in cirrhosis, often even detected incidentally since HCC was suspected and led to the treatment recommendation of ablation. Therefore, most data are of a retrospective nature, analyzing the results after incidentally detected and already treated tumors. 

Not much is known about the efficacy of MWA for the treatment of primary ICCA in the healthy liver or for the treatment of recurrent disease, in particular because these tumors are often diagnosed at advanced stages and treated by major liver resections or systemic therapy [42]. Since ablation is still limited to tumors <3 cm, with some studies now showing efficacy for the treatment of HCCS 3–5 cm [43], the role for the primary treatment of ICCA in the healthy liver is rather negligible. Patients with recurrent ICCA in the healthy liver are mostly treated by chemotherapy or re-resection, and ablation is usually only recommended in individual cases discussed in the tumor board. A potential advantage is the fact that ablation can be carried out while chemotherapy is performed. Next generation sequencing (NGS) is currently changing the treatment regime for ICCA with new treatments for the recently detected mutations IDH1 and FGFR2 (Ivosidenib and Pemigatinib) showing promising results [44]. If these modern treatment approaches based on NGS continue to show their efficacy in ICCA patients [3] and if an additive treatment by ablation improves, the outcome still needs to be analyzed. Patients with recurrent disease in cirrhosis often have limited systemic treatment options due on the underlying liver disease [45] and might therefore be ideal candidates for a treatment with ablation.

The role for the combination of ablation with embolization therapy for ICCA still needs to be explored and most likely depends on the underlying tumor biology. Some initial reports suggest that there might be role for this approach in tumors >3 cm and inoperable or unresectable patients [39].

As known from HCC and other liver tumors routinely treated by ablation, the use of image-guided computer-assisted navigation is safe and efficient and helps to increase the number of treatable tumors by facilitating ablation in difficult anatomic locations of the liver, for tumors close to important vasculature or adjacent organs or even those invisible on ultrasound and CT [19]. In addition, these technical advancements optimize the treatment by including an ablation planning and validation software allowing doctors to precisely plan the ablation zone and to immediately validate the results in the control scan during the intervention. While there are only very limited data available on the use of computer-assisted image-guided navigation for the treatment of ICCA [40], we can herein report our initial experience with 10 patients with SMWA showing that this approach is safe and efficient and might be a treatment alternative to resection or systemic treatment in individual cases. Clearly more data are urgently needed and indications for primary ICCA in the cirrhotic liver, primary ICCA in the healthy liver and ICCA recurrence or their metastases, should be analyzed separately.

## 5. Conclusions

Although prospective data of ICCAs treated by ablation and in particular SMWA are still lacking, this minimally invasive and tissue-sparing therapeutic option might be a safe and efficient treatment alternative for small primary ICCA as well as for metastatic ICCA in individual patients.

## Figures and Tables

**Table 1:** Summary of the literature on MWA for ICCA.

Reference of Original Publication	Year of Publication	Technique(s) Analyzed	Number of Patients Included	Number of Treated Lesions	Size (Range; cm)	OS 1, 3, 5 Years (%)	RFS 1, 3, 5 Years (%)
[26]	2011	MWA	15	24	3.2 (1.3–9.9)	60, -, -	-
[23]	2012	MWA	18 (6)	25	2.5 (0.7–4.3)	36.3, 30.3, 30.3	-
[27]	2015	MWA/TACE	26	39	3.6 (2.5–6.5)	69.2, 61.5, -	-
[28]	2018	MWA	107	171		93.5, 39.6, 7.9	41.5, -, -
[29]	2018	RFA vs. MWA	20	50	1.8 (0.5–4.7)	-	-
[30]	2019	RFA vs. MWA	71 (35)	98 (50)	3.6 ** (2.2–7.2)	95, 75, 68 **	79, 59, 55 **
[31]	2019	MWA	78	106	3.1 (0.8–5)	78.9, 52.2, 35	78.9, 19.9, -
[32]	2019	MWA vs. surgery	121 (56)	136 (62)	2.7 (0.8–5)	81.2, 42.5, 23.7 **	70.3, 33.1, - **
[33]	2020	MWA/TACE	275 (92)	-	5.7	50, 21.5, 6.1	41.1, 21.5, 6.1
[34]	2020	MWA	221 {32}	285	-	-	-
[35]	2020	MWA/RFA	27 (3) *	33	2.1 (2.0–2.8)	88.9, 40.7, 14.8	-
[36]	2021	MWA	52	74	3.1 (0.8–5)	87.4, 51.4, 35.2	68.9, 56.9, 56.9

MWA = microwave ablation; RFA = radio radiofrequency ablation; TACE = transarterial chemoembolization; OS = overall survival, RFS: recurrence free survival. * Results for different ablation, not separated; ** Results for MWA; () = CCA; { } = ICCA.

**Table 2:** Patients with primary ICC treated by SMWA.

Patient	Age at SMWA	Gender	Child Score	Underlying Liver Disease	Segment	Tumor Size (mm)	Previous Treatment	Complications > Dindo IIIa	Local Recurrence	Time to Recurrence (Months)	Treatment of Recurrence	Death	OS (Months)	Time to Transplant (Months)	Overall Disease Progression
1	31	M	B7	PSC	V	20	no	no	no			no	31.5	10	no
2	58	M	A6	HCV	VIII	23	no	no	yes	18	TAE + MWA planned	no	19	N/A	no
3	64	M	A6	ASH/NASH	VIII	15	TAE	no	no			no	12	N/A	no
4	58	M	A5	NASH	VI	24	no	no	no			no	10	on waiting list	yes (HCC)
5	81	M	A5	Unknown	V/VIII	27	No	no	no			no	6		no

Gender: M = male, F = female; OS = overall survival; HCV = hepatitis C; PSC = primary sclerosing cholangitis; TAE = transarterial embolization; ASH = alcoholic steatohepatitis; NASH = non-alcoholic steatohepatitis.

**Table 3:** Patients with ICC metastases treated by SMWA.

**Patient**	**Age at SMWA**	**Gender**	**Tumor Stage**	**Previous Treatment**	**Adjuvant CTX**	**Location Metastasis Segment**	**Size of Ablated Metastasis**	**Complications > Dindo IIIa**
1	72	W	T2aN1M0 L0V0Pn1 G1 R0	ALPPS	Gemcitabine	II	32	no
2	37	W	T1N0M1 L0V0Pn0 R0 G3	Right hepatectomy + metastectomy left liver	Xeloda	II	15	no
3	58	M	T1N0M0 Ln1V0Pn0R1 G3	ALPPS	Gemcitabine/ Capecitabine	IVb	26 & 25	no
4	66	W	T2N0M0 Ln0V1Pn0R1 G2	Extended left hepatectomy	none	VIII	17	no
5	56	M	T2aN0M0 Ln0V0Pn1R0 G1	ALLPS	none	III	6	abscess
**Patient**	**Local Recurrence**	**Time to Recurrence (Months)**	**Treatment of Recurrence**	**Death**	**OS after SMWA (Months)**	**OS after** **Initial Diagnosis**	**Overall Disease Progression**	
1	yes	5	SBRT	yes	19	45	yes	
2	no			no	20	34	no	
3	yes	3	CTX	yes	5.5	35	yes	
4	no			yes	6.5	15	no	
5	no			no	1	25	no	

Gender: M = male, F = female; OS = overall survival; CTX = chemotherapy; SBRT = stereotactic body radiotherapy; ALPPS = Associating Liver Partition and Portal Vein Ligation.

## Data Availability

Data are available on the request.
